# Development of the Behavioral Inflexibility Scale for Children with Autism Spectrum Disorder and Other Developmental Disabilities

**DOI:** 10.1002/aur.2257

**Published:** 2020-01-06

**Authors:** Luc Lecavalier, James Bodfish, Clare Harrop, Allison Whitten, Desiree Jones, Jill Pritchett, Richard Faldowski, Brian Boyd

**Affiliations:** ^1^ The Ohio State University Columbus Ohio; ^2^ Vanderbilt University Nashville Tennessee; ^3^ University of North Carolina at Chapel Hill Chapel Hill North Carolina; ^4^ University of Texas at Dallas Richardson Texas; ^5^ University of Kansas Kansas City Kansas

**Keywords:** autism spectrum disorder, measurement, outcome, behavior inflexibility, development, repetitive behavior

## Abstract

Behavior inflexibility (BI) refers to rigid patterns of behavior that contrast with the need to be adaptable to changing environmental demands. We developed a parent‐reported outcome measure of BI for children with autism spectrum disorder (ASD) and other developmental disabilities with a multi‐step iterative process. A pool of 62 candidate items was generated through expert panel feedback, review of existing scales and focus groups. A consensus process was used to generate the final 38 items. Parents of 943 children (age range, 3–18 years; average, 11.4 years; 79% boys) with ASD completed an online survey. One hundred thirty‐three parents rated their child twice within 3 weeks (average = 16.5 days). A series of factor analyses suggested that the 38 items measured a single construct. Scores had a weak correlation with level of functioning (−0.12) and did not differ based on sex. Scores had a negligible correlation with age (−0.07), although measurement invariance was not supported. The mean total score for the Behavioral Inflexibility Scale (BIS) was normally distributed. Internal consistency was *α* = 0.97 and temporal stability was *r* = 0.92. Correlations with parent ratings on the subscales of the Repetitive Behavior Scale—Revised varied from 0.48 to 0.89. The correlation with parent ratings on the Social Communication Questionnaire total score was 0.52. Our data show that BI in children with ASD ranges significantly from mild to severe and that the 38‐item BIS is valid and reliable. ***Autism Res** 2020, 13: 489–499*. © 2020 The Authors. *Autism Research published by International Society for Autism Research* published by Wiley Periodicals, Inc.

**Lay Summary:**

We developed a parent‐completed rating scale of behavior inflexibility (BI) for children with developmental disabilities using a multistep process. The Behavioral Inflexibility Scale (BIS) contains 38 questions rated on a 6‐point scale. Parents of 943 children with autism spectrum disorder (ASD) completed an online survey. We examined associations between the BIS and other scales and demographic variables. The BIS is valid and reliable. BI in children with ASD ranges from mild to severe.

## Introduction

There is a clear consensus in the field of autism research on the need to develop psychometrically sound outcome measures [Anagnostou et al., 2014; Lecavalier, Wood, Halladay, Jones, & the Autism Speaks clinical workgroup, 2014; Scahill et al., 2015]. This is seen as a rate‐limiting step to the identification of interventions that effectively treat those persons whose autism spectrum disorder (ASD) is associated with some degree of functional impairment and for whom intervention is sought. The core symptoms of ASD represent obvious outcome measurement targets; yet, there continues to be a paucity of measures that are sensitive to change [Anagnostou et al., 2014; Scahill et al., 2015].

The purpose of this study was to develop a psychometrically sound and dimensional outcome measure of what we term “behavioral inflexibility” (BI). BI is here meant to refer to rigid and inflexible patterns of behavior that contrast with the need to be flexible, open to change, and amenable during situations that are unpredictable and require more adaptive responding. We chose to focus specifically on BI for several reasons: (a) in his original account of autism, Kanner [1943] specifically referred to inflexibility as being a defining feature of the condition. (b) Previous studies have examined the latent factors into which the variety of discrete repetitive behaviors can be subtyped in the context of ASD, and across these studies that differ considerably in measurement method and sample age range, the factor associated with insistence on sameness, inflexibility, and resistance to change or novelty has consistently been isolated [Bishop, Richler, & Lord, 2006; Cuccaro et al., 2003; Hus, Pickles, Cook, Risi, & Lord, 2007; Lam, Bodfish, & Piven, 2008; Szatmari et al., 2005]. (c) Inflexibility is associated with a wide range of domains of functioning in ASD, including cognition [e.g., cognitive flexibility or set shifting, (Miller, Ragozzino, Cook, Sweeney, & Mosconi, 2015)] rumination or repetitive thinking [Gotham, Bishop, Brunwasser, & Lord, 2014], language [Muskett, Perkins, Clegg, & Body, 2009] (e.g., perseverative language, echolalia), social function [Christ, Holt, White, & Green, 2006], play [Honey, Leekam, Turner, & Mcconachie, 2006] and eating/mealtime [Johnson et al., 2014]. (d) Sameness/inflexibility is also positively associated with the severity of comorbid psychiatric symptoms that are common in ASD including anxiety [Duvekot, Ende, Verhulst, & Greaves‐Lord, 2017; Uljarević, Richdale, Evans, Cai, & Leekam, 2017], depression [Gotham et al., 2014], and hyperactivity [Gabriels, Cuccaro, Hill, Ivers, & Goldson, 2005]. These findings are consistent with evidence that behavioral and cognitive inflexibility are prominent features of related neuropsychiatric disorders, such as Obsessive‐Compulsive Disorder (OCD), Attention Deficit Hyperactivity Disorder (ADHD), Depression, and Anxiety Disorder [Geurts, Corbett, & Solomon, 2009], which is in line with the current Research Domain Criteria “transdiagnostic model” that characterizes different neurodevelopmental conditions as sharing a set of common underling symptom domains [Kozak & Cuthbert, 2016].

Relevant to ASD, it is likely that BI can account for several of the clinically expressed restricted and repetitive behaviors (RRBs) observed in the disorder. Furthermore, a number of instruments have been developed and used to study RRBs in the context of ASD [see Honey, Rodgers, & McConachie, 2012 for a review]. Existing RRB instruments, such as the Repetitive Behavior Scale—Revised (RBS‐R) [Bodfish, Symons, Parker, & Lewis, 2000] and the Repetitive Behavior Questionnaire [Honey, McConachie, Turner, & Rodgers, 2012], focus primarily on delineating specific topographies and also the frequency of these behaviors. These instruments have proven to be quite useful in studying the phenomenology of RRBs but as noted by the an expert panel convened by Autism Speaks, all have at least some limitations as treatment outcome measures [Scahill et al., 2015, 2019]. In addition, the aforementioned instruments were not developed to measure the degree of functional impact associated with these behaviors. The RRB instruments that attempt to capture functional impairment, such as the ASD version of the Child Yale‐Brown Obsessive Compulsive Scale [Scahill et al., 2006], Sameness Questionnaire [Prior & MacMillan, 1973], Self‐injury Trauma Scale [Iwata, Pace, Kissel, Nau, & Farber, 1990], and Interests Scale [Turner‐Brown, Lam, Holtzclaw, Dichter, & Bodfish, 2011], also were not developed as outcome measures and tend to focus on only a specific, singular form of repetitive behavior as opposed to measuring the full range of these behaviors commonly seen in individuals with ASD and related neurodevelopmental conditions. The paucity of valid instruments that actually measure the functional impact associated with the broad variety of RRBs observed in ASD leads to difficulties identifying appropriate treatment cases and targets.

Like all aspects of the ASD phenotype, the pattern of expression of RRBs, and the level of severity associated with these behaviors, is highly variable across individuals. For example, “lower‐order” RRBs such as stereotyped hand‐flapping or body rocking are often benign and therefore not specifically targeted for intervention. However, in a subset of cases, interruption of these actions can lead to emotional outbursts and more severe behavior challenges (e.g., aggression, self‐injury) that do become the target of treatment [Richards, Oliver, Nelson, & Moss, 2012; Rojahn, Barnard‐Brak, Medeiros, & Schroeder, 2015]. Likewise, “higher‐order” RRBs, such as insistence on sameness or circumscribed interests have been reported to be adaptive or a source of resilience (e.g., routines as a way to deal with unpredictability, intense interests as a way to make friends or develop vocational skills). Yet, in some circumstances the opposite is true and rigidly held routines or overly narrow interests lead to avoidance, restriction of experience, and stress within the larger family, school, or community context [Gotham et al., 2014; Turner‐Brown et al., 2011; Uljarević et al., 2017]. As these clinical scenarios show, there is a subset of individuals with ASD whose RRBs are associated with a clinically significant degree of functional impairment. From the perspective of treatment development and outcome measurement, it is the goal of intervention studies to identify and treat this subset of cases whose RRB is actually leading to functional impairment.

To summarize, while existing RRB instruments provide psychometrically sound ways to identify the presence or absence of RRBs and also examine phenomenology, they are insufficiently sensitive to the measurement of functional impairment and to changes in impairment over time.

To respond to the needs of the field for more robust and clinically meaningful outcome measures, we report on the development of a new parent‐rated measure of BI, the Behavioral Inflexibility Scale (BIS). The BIS was developed to measure the severity and impact of BI on the everyday activities of families of children with ASD (and other neurodevelopmental disorders) as well as demonstrate sensitivity to change over time. Here we describe the process we used for item generation and item reduction that produced the final version of the BIS. We also describe results of the psychometric analyses of the BIS using data from a large sample of children with ASD.

## Method

This research was prospectively reviewed and approved by the appropriate Institutional Review Board.

### Item Development and Reduction Procedure

A seven‐step process was used to develop the final pool of items used for data collection and analysis. The process included the following.

#### Development of construct

We began by developing a working definition of BI (“resistance to changing or adapting one's behavior in line with a changing situation”) as the guiding concept for scale development and by identifying six potential dimensions that could help to define this construct more objectively (response to change, response to novelty, response to uncertainty, engagement in routines, tendency to be restrictive or picky, and oversensitivity to ambient events).

#### Expert panel review

We solicited feedback on this definition and set of dimensions from a panel of ten ASD experts. The experts were recruited based on their experience in ASD clinical research in a variety of areas such as repetitive and sensory behaviors, social‐communication, measure development, and intervention. Expert feedback was used to refine the working definition and to reduce the set of dimensions to five (oversensitivity was not deemed particularly relevant to the BI construct).

#### Caregiver and clinician focus groups

We used purposive sampling to recruit focus group participants to solicit feedback representing a range of child age (preschool and school aged), biological sex, and cognitive / language ability. Four caregiver groups (*n* = 6–9 per group for a total of 30 parents), and three clinician groups (*n* = 5–11 per group for a total of 25 clinicians) were recruited from three different sites (North Carolina, Ohio, Tennessee). Trained moderators led the groups using a focus group guide that was based on the working definition and set of dimensions derived from the expert panel feedback. All sessions were audiotaped and transcribed verbatim. Overall, caregivers and clinicians expressed very similar insights regarding the manifestation, impact, and strategies to manage BI. One caregiver transcript was coded using a semantic coding approach by all raters from each site. The semantic code set was then reduced to a subset of the codes used most consistently across raters. All caregiver and clinician transcripts were then coded independently by two coders using this common semantic code subset. Frequency of codes across all transcripts was analyzed using a Thematic Analysis approach [Braun & Clarke, 2006] to derive a set of 21 themes that consistently emerged from both the caregiver and clinician focus groups (e.g., restricted engagement in activities, development of routines or rituals, impact on social functioning, family accommodation, child stress, caregiver stress). More information on the focus group process and results is available in a previous paper by our group [Sethi et al., 2018].

#### Review of existing measures

We selected a set of rating scales from the literature and all items from these scales were rated independently by two raters. Thirteen scales were selected based on five constructs (repetitive behaviors, sensory behaviors, executive function/cognitive flexibility, temperament, and social‐communication behaviors). A total of 343 items from the five scales were reviewed and rated by two independent raters on (a) whether the item fit the working definition of BI (yes or no), and (b) if yes, then with what degree of confidence (1 [lowest] to 4 [highest]). Sixty‐two items were selected based on multiple rater agreement and highest confidence rating as relating to the BI construct (mean inter‐rater agreement across all items was 85.1%).

#### Developing the initial item pool

The authors used a consensus approach to link the 62 items to the themes that emerged from the thematic coding of the focus group transcripts. Items within each theme that were judged to be redundant were dropped resulting in a pool of 42 items. These 42 items were rewritten by the authors in an effort to best fit the working definition of BI within each theme. Response options (6‐point Likert scale) and time frame (1 month) were selected to optimize measurement of change.

#### Expert panel review to reduce to final item pool

The 42‐item set was independently reviewed by each of the ten expert panel members to determine appropriateness of the items with respect to our working definition and to solicit suggestions for item wording (mean percent agreement across raters for all items = 85%). This reduced our item pool to a final set of 38 items.

#### Cognitive interviewing

We administered the 38‐item scale using a cognitive interviewing approach [Campanelli, 1997; Willis, Royston, & Bercini, 1991] to a group of eight parents/caregivers of children with ASD to arrive at the final wording for each item. Cognitive interviewing is a one‐to‐one interview technique where verbal probing techniques are used to elicit respondent thinking about survey and interview questions. It helps ensure respondents are interpreting items and response options in a uniform way. Cognitive interviews used a standardized protocol of probes developed by a qualitative researcher. Interviews were transcribed and coded for common themes. This final step did not end up reducing the number of items, but led to further wordsmithing to ensure parents understood item intent and meaning.

### Participants for Online Survey

Participants were the caregivers of 943 children with ASD. Participants were recruited via the Interactive Autism Network (IAN) Research Database at the Kennedy Krieger Institute, Baltimore, MD (https://iancommunity.org). IAN is an online registry of individuals with ASD. It was developed to support internet‐based recruitment efforts for research studies. Interested parents on the IAN registry who indicated willingness to complete online research surveys received an email notice about the survey. IAN relies upon parent report of a child's clinical diagnosis, however, also includes ratings from the Social Communication Questionnaire (SCQ) [Rutter, Bailey, & Lord, 2003], Social Responsiveness Scale [Constantino & Gruber, 2005], Autism Diagnostic Observation Schedule (Lord, Rutter, DiLavore, & Risi, 2002), and Autism Diagnostic Interview—Revised [Rutter, LeCouteur, & Lord, 2003]. The IAN registry has been used extensively within studies of this nature [e.g., Scahill et al., 2019] and clinical information reported by parents has been found to be valid [Daniels et al., 2012].

In order to avoid undue selection bias and to insure that we broadly sampled the full range of inflexibility manifestation in the context of ASD, in the survey introduction, we stated that the study's purpose was to examine “patterns of behavior in children with ASD.” A total of 11,337 families with children and adolescents between the ages of 3 and 17 years of age inclusive were contacted. A total of 1,135 clicked on the link for our survey. Of those, 943 completed the survey. Demographic characteristics of the caregivers and youth with ASD are presented in Table 1.

**Table 1:** Demographic and Clinical Characteristics of Participants in Online Survey (*N* = 943)

	*N*/average (SD)	%
Age	11.4 (4.0)	
Gender		
Males	749	79.4
Females	194	20.6
Caregiver race		
White	846	89.7
Black	40	4.2
Other	57	6.0
Parent‐reported diagnoses		
Autism spectrum disorder	940	99.7
ADHD	386	40.9
Anxiety	345	36.6
No comorbid diagnosis	337	35.7
Parent‐reported child IQ		
Above average	267	28.3
Average	251	26.6
Below average	272	28.9
Unknown	153	16.2
Parent‐reported verbal ability		
No meaningful words	58	6.2
10+ words, no 2–3 word sentences	85	9.0
Consistently uses 2–3 word sentences	143	15.2
Complete sentences most of the time	657	69.7
Educational placement		
General education	207	22.0
Special education	305	32.3
General education and special education	356	37.8
Home school	70	7.4
Other	5	0.5
Caregiver education		
Some high school	3	0.3
High school graduate or GED	57	6.0
Some college	242	25.7
College graduate	348	36.9
Advanced degree	293	31.1
Household income (*n* = 936)		
≤$20,000	61	6.5
$20,001–$40,000	125	13.4
$40,001–$60,000	159	17.0
$60,001–$90,000	185	19.8
$ $90,001+	406	43.4
Social Communication Questionnaire (SCQ) (*n* = 941)	17.8 (6.7)	
SCQ < 12	172	18.3
SCQ ≥ 12	769	81.7

### Measures Used in Online Survey

#### Behavioral Inflexibility Scale

The BIS is a 38‐item caregiver completed scale. Items are rated on a 6‐point rating scale ranging from “Not at all a problem” [0] to “Very severe or Extreme problem” [5]. Raters assess behaviors over the past month.

#### Social Communication Questionnaire

The SCQ [Rutter et al., 2003] consists of 40 items arranged onto three subscales: social interaction, communication, and stereotyped behaviors. Studies have suggested that a total score greater or equal to 12 can be ideal to maximize sensitivity [Norris & Lecavalier, 2010]. There are two versions to the SCQ. The Lifetime version considers the child's entire developmental history, while the Current version focuses on the child's behavior over the last 3 months. This study used the Current version. In the current sample, the internal consistency was 0.83 for verbal children (*n* = 816) and 0.80 (*n* = 127) for nonverbal children.

#### Repetitive Behavior Scale—revised

The RBS‐R [Bodfish et al., 2000] is a 43‐item caregiver report measuring a variety of repetitive behaviors. Items are rated on a 4‐point Likert scale and distributed along six subscales: Stereotyped Behavior, Self‐Injurious Behavior, Compulsive Behavior, Ritualistic Behavior, Sameness Behavior, and Restricted Behavior. In the current sample, internal consistency for the subscales ranged from 0.74 (ritualistic behavior) to 0.90 (sameness behavior).

### Test Retest Procedure

For the initial survey, parents were asked to complete the 38 BIS items, a demographic form, and the SCQ. Twenty percent of parents were invited to complete the BIS a second time as well as the RBS‐R between 2 and 3 weeks after the initial ratings to assess temporal stability (*n* = 133) [age range, 3–17.8 years; average, 11.0 (SD: 4.0) years; 79% boys]. This sample did not differ statistically from the overall sample on any of the demographic characteristics.

### Analytical Plan

An iterative approach was used to understand the structure underlying the 38 items. The full sample was randomly divided into a calibration sample (*n* = 471) and a validation sample (*n* = 472). We used exploratory factor analysis (EFA) and confirmatory factor analysis (CFA) on the calibration sample to assess and validate the scale's structure, and obtain indices of model fit. We then applied this sample model to the validation sample to verify the results. Model fit was evaluated with the commonly used indices: root mean square error of approximation (RMSEA), comparative fit index (CFI), Tucker–Lewis index (TLI), and standardized root mean square residual (SRMR). Guidelines to evaluate whether a given model provided a good approximation to the data included: RMSEA and SRMR less than 0.1 and CFI and TLI greater than 0.9 [Browne & Cudeck, 1992; Hu & Bentler, 1999]. We also evaluated measurement invariance for children of three different age bands (preschool and kindergarten [3 ≤ age < 6], elementary school [6 ≤ age < 12], and junior high school/high school [12 ≤ age < 18]). Measurement invariance refers to the degree to which psychometric properties of a set of items are comparable across groups. In other words, when measurement invariance holds, a set of items is measuring a construct in the same way for the different groups (child's age in this case).

Factor analysis models were estimated employing mean and variance adjusted weighted least squares estimators with robust standard errors [DiStefano & Morgan, 2014; Muthén, 1984; Muthén & Muthén, 1998–2017; Wirth & Edwards, 2007]. All factor analyses were based on the polychoric correlations between the items. All analyses were conducted using the SAS [SAS Institute Inc., 2013], MPlus [Muthén & Muthén, 1998–2017], and FACTOR [Ferrando & Lorenzo‐Seva, 2017b; Lorenzo‐Seva & Ferrando, 2019] software packages. All factor analysis models were identified by setting latent factor means to 0 and latent factor variances to 1, such that all item factor loadings and residual covariances are estimatable under the MPlus “delta” parameterization [Muthén & Asparounov, 2002; Muthén & Muthén, 1998–2017]. In the measurement invariance analyses by age group, estimation was conducted under an MPlus “theta” parameterization [Muthén & Asparounov, 2002; Muthén & Muthén 1998–2017], which allows for separate estimation of factor variances, factor loadings, and residual variances between age groups.

Internal consistency was measured with Cronbach's alpha and temporal stability was estimated with intraclass correlations (ICC) for absolute agreement under a generalizability theory framework. Associations between BIS and SCQ and RBS‐R were assessed with Pearson's correlations. Convergent validity was assessed by examining correlations with the RBS‐R and repetitive behavior subscale of the SCQ, while divergent validity was assessed by examining the correlation with the social‐communication subscale of the SCQ.

## Results

### Factor Analyses and Assessment of Dimensionality

EFAs on the interitem polychoric correlation matrix suggested a one‐factor solution, with one dominant eigenvalue that accounted for over 56% of the total item variance and a magnitude more than 13 times larger than the second eigenvalue. Solutions with two to eight factors were considered, but factors beyond the dominant factor had little clinical meaningfulness. In addition to the scree plot [Cattell, 1966], the Hull method, item and global unidimensional congruence indices, item and global explained common variance indices, and item and global residual absolute loadings indices all suggested a unidimensional solution [Lorenzo‐Seva, Timmerman, & Kiers, 2011; Ferrando & Lorenzo‐Seva, 2017b]. Specifically, the global unidimensional congruence index was 0.99 (exceeding the criterion of 0.95); the global explained common variance was 0.94 (exceeding the criterion of 0.85); and the mean of item residual absolute loadings was 0.15 (which is lower than the cutoff threshold of 0.30) [Ferrando & Lorenzo‐Seva, 2017a, 2017b; 2018]. Moreover, only a small fraction of individual items showed deviations from item‐level unidimensionality criteria, and when this occurred, the deviations were small in magnitude.

In contrast to the strong indications of unidimensionality from the aforementioned indices, the factor analytic fit indices suggested some misfit. In an attempt to better understand the sources of model misfit, two additional sets of analyses were undertaken: bifactor solutions [Morin, Arens, & Marsh, 2016] and CFA models with correlated residuals. Bifactor solutions (analytic details not shown, but available upon request) suggested slightly improved model fits, but the improved model fit was achieved due to additional minor factors that partially reflected the conceptual domain structure built into the items, as well as slight variations in item wording and content. We selected 12 pairs of items with the highest modification indices for the CFA with correlated residuals. These analyses yielded slightly improved model fit. The correlation between loadings from the 1‐factor model with and without correlated residuals was *r* = 0.993 in the Development sample and *r* = 0.994 in the Validation sample. Thus, although the inclusion of correlated residuals improved the model fit, the inclusion of correlated residuals did not have a substantive impact on the factor analytic results.

Table 2 shows fit indices for EFAs and CFAs with and without correlated residuals. Table 3 shows summary items and factor loadings with correlated residuals for the full sample. All loadings were statistically significant at *p* ≤ 0.0001.

**Table 2:** Indices of Fit for EFA and CFA

Sample/model	*χ* ^2^	df	RMSEA (90%CI)	CFI	TLI	SRMR
Exploratory (*n* = 471)						
Single factor	3256	665	0.091 (0.088–0.094)	0.938	0.934	0.050
Single factor with CR	2294	653	0.073 (0.070–0.076)	0.960	0.957	0.043
Confirmatory (*n* = 472)						
Single factor	3561	665	0.096 (0.093–0.099)	0.925	0.920	0.055
Single factor with CR	2810	653	0.084 (0.080–0.087)	0.944	0.939	0.050
Full (*n* = 943)						
Single factor	6906	665	0.100 (0.098–0.102)	0.920	0.916	0.050
Single factor with CR	4941	653	0.083 (0.081–0.086)	0.945	0.941	0.043

**Table 3:** Summary Items and Factor Loadings in Descending Order of Magnitude

23. Trouble tolerating changes to daily routine	0.895
36. Insists that the order of events or activities stay the same	0.894
20. Needs things to remain the same	0.876
34. Family has to maintain a consistent routine	0.860
26. Bothered by changes in plans	0.847
32. Gets upset by changes that seem minor to others	0.847
15. Reacts negatively when unexpected things happen	0.841
14. Family changes the way does things	0.832
35. Very particular way of doing most things	0.830
37. Dislikes when things are unpredictable	0.818
6. Dislikes changes to his surroundings	0.812
18. Family needs extra time to get things done	0.805
25. Family avoids trying new things	0.802
38. Trouble tolerating new experiences	0.799
30. Wants to complete specific routines or rituals	0.794
22. Prefers to do things the same way	0.778
28. Insists that other people do things in a certain way	0.766
31. Has rigid or routine ways in play or leisure	0.759
19. Insists that certain items or objects are available	0.758
1. Resists having to change the way he does things	0.755
17. Becomes upset if interrupted	0.751
8. Difficulty transitioning between activities	0.747
4. Must instruct others how to interact with child	0.742
10. Family limits community outings	0.733
9. Hard to redirect from things he is doing	0.724
2. Takes a long time to get comfortable in new situations	0.722
7. Has trouble coming up with new ways of doing things	0.704
24. Has to keep things in the same place	0.699
16. Insists use specific routes	0.688
27. Has trouble leaving play or leisure activities	0.686
21. Reluctant to try new things	0.659
11. Dislikes changes in the appearance of others	0.648
13. Difficulty interacting with peers	0.617
3. Gets stuck on particular activities or topics	0.606
33. Prefers to stick with one topic or activity	0.604
29. Insists on wearing certain items of clothing	0.571
12. “Rule‐driven” or “rule‐bound”	0.481
5. Insists on eating certain foods at mealtimes	0.471

*Note*. Correlated residuals were between Items 1 and 2 (0.15), 10 and 25 (0.20), 10 and 14 (0.18), 8 and 9 (0.27), 9 and 27 (0.21), 17 and 27 (0.18), 12 and 13 (0.22), 13 and 33 (0.21), 3 and 33 (0.27), 37 and 38 (0.12), 21 and 38 (0.15), and 5 and 21 (0.21).

Measurement invariance across age groups (preschool/kindergarten, elementary school, junior/senior HS) suggested that the fit of the configural invariance model was marginal according to standard CFA fit indices, but the single‐factor model clearly emerged within each age group when analyzed separately. Comparing a metric invariance model (equality of factor loadings) to the 1‐factor configural invariance model led to clear rejection of the metric invariance hypothesis (*χ* ^2^(74) = 143.24; *p* ≤ 0.00001). The primary reason for the lack of fit seems to lie in differential relevance of BIS items for children of different ages, with about 16 BIS items showing modest, but statistically significant negative correlations between item scores and children's ages.

**Figure 1:**
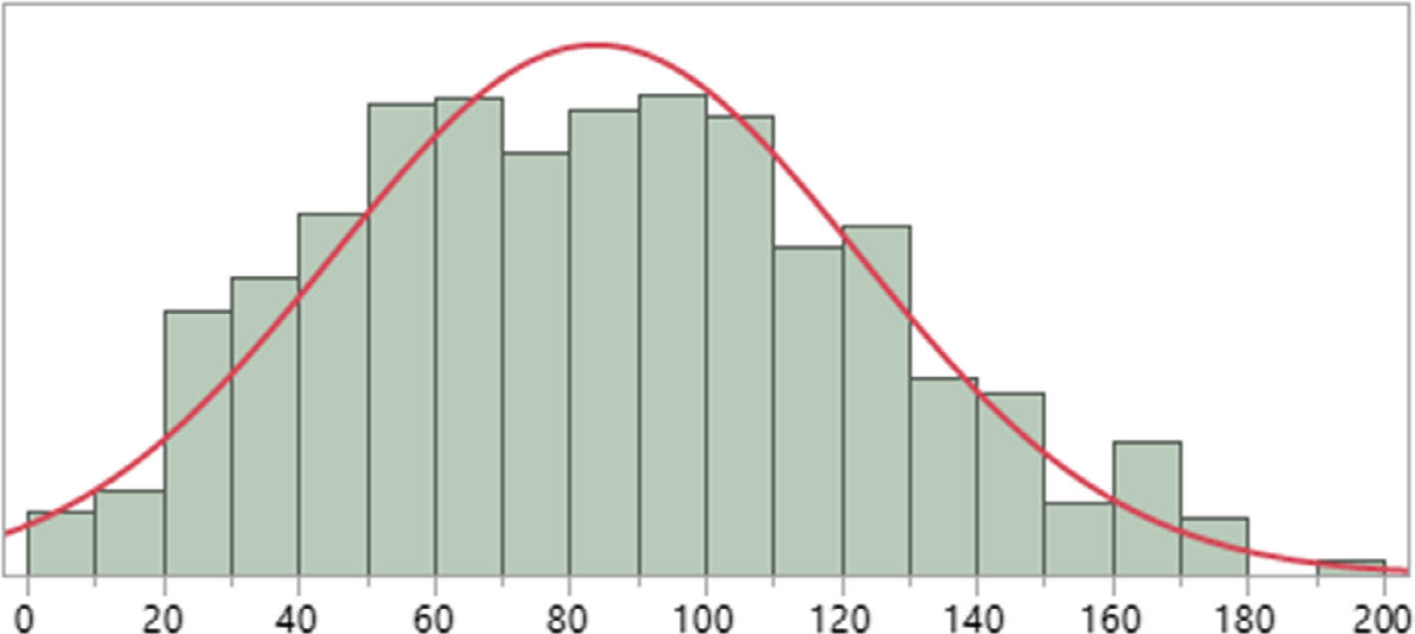
Distribution of total scores on BIS (*N* = 943).

Figure 1 shows distribution of the BIS scores. Scores were normally distributed and had an average of 83.9 and SD of 38.6 (range 2–190).

### Reliability and Validity

Cronbach's alpha is a measure of the degree of homogeneity of responses and provides a lower‐bound estimate to the measure's reliability for a specific population. The Cronbach's alpha for the BIS scale was 0.97. Similarly, test–retest reliability (average = 16.5 days) for a randomly selected subset of 133 cases was ICC = 0.92, suggesting high temporal stability.

Table 4 shows relationships between total BIS score and demographic and clinical variables from the online survey. The mean BIS score had negligible correlations with demographic characteristics. It had a moderate positive correlation with the SCQ and most RBS‐R scores. The correlation with the RBS‐R Sameness subscale was especially high at *r* = 0.89.

**Table 4:** Correlations with demographic and clinical variables

Child age (*n* = 943)	−0.07
Level of functioning	−0.12*
Parent‐reported child verbal ability	−0.08
SCQ (*n* = 941)	
SCQ total	0.52**
SCQ Social Interaction (*n* = 943)	0.36**
SCQ Communication (*n* = 938)	0.33**
SCQ RRB (*n* = 939)	0.51**
Repetitive Behavior Scale—Revised (RBS‐R; *n* = 133)	
RBS‐R total	0.84**
Stereotypy	0.52**
Self‐injury	0.48**
Compulsive	0.68**
Ritualistic	0.73**
Sameness	0.89**
Restricted	0.61**

*P ≤ 0.01; **P ≤ 0.0001.

## Discussion

We used a multimethod approach to develop a dimensional measure of BI. Following this, psychometric evaluation of the final 38‐item pool indicated (a) that the instrument provides a unidimensional set of items that measure inflexibility, (b) yields an approximately normal distribution for the overall score in a large sample of children with ASD, (c) has strong concurrent validity with an independent measure of RRBs, and (d) appears to vary independent of child age, sex, and parent reported cognitive and language ability in this sample.

We found that the 38‐item BIS resolves into a single overall “inflexibility” factor. This is a desirable feature of a potential intervention outcome measure because it can diminish data collection burden and contribute to more parsimonious interpretation of results. This is in contrast to a number of existing measures of RRBs that have been shown to yield multidimensional solutions [Bodfish et al., 2000; Honey, McConachie, et al., 2012; Scahill et al., 2006]. Although previous studies using these measures have utilized a total or overall RRB score, this approach can be questioned when the measure resolves into distinct dimensions or factors. Thus, one potential advantage of the BIS is that it may be tapping successfully a single construct that potentially underlies many different subtypes of repetitive behaviors. We also found that the BIS total score was normally distributed in our large and diverse ASD sample. This suggests that inflexibility, as measured by the BIS, is a dimensional construct in the context of ASD. This is a desirable feature of a potential intervention outcome measure because it supports the use of a single‐dimensional score of impairment to select the subset of cases that meet a specified *a priori* criteria for entry into treatment studies. A similar approach has been used to considerable success in the treatment of irritability in ASD, where a dimensional measure derived from the Aberrant Behavior Checklist (ABC) irritability subscale [Aman & Singh, 2017] has been used to specify clinically significant levels of impairment for study entry [Aman et al., 2009; Bearss et al., 2015; Handen et al., 2015].

We explored further item reduction and concluded that it was not justified. The average interitem correlation was 0.55 (ranged = 0.19 to 0.82). In fact, only 2 of the 703 correlations were above 0.80. Deleting items had virtually no impact on internal consistency. Perhaps more importantly than the statistical arguments, we believed item content was sufficiently unique to retain all 38 items.

An important point with respect to dimensionality of inflexibility that we did not address in our study is the relation between ASD and either typical development or other concurrent clinical comorbidities such as ADHD. At this point we do not know if inflexibility as measured by the BIS is normally distributed in the general population with individuals with ASD falling at the more severe end of the dimension in a continuously distributed fashion (e.g., extreme ends of “normal” inflexibility) or in a discontinuous fashion (e.g., a bimodal distribution). In an on‐going study of the BIS comparing several clinical and nonclinical groups we will begin to address this question, but clearly more work examining the dimensionality of inflexibility in a variety of clinical groups is needed.

Finally, we found that the BIS total score in our sample of children with ASD varied independently of both child age and child sex. In addition, the BIS total score in our ASD sample was not related to either parent‐reported cognitive ability or language level of their child. Measurement invariance across age groups suggested that the fit of the configural invariance model was marginal according to conventional CFA fit indices, but the single‐factor model clearly emerged as the most tenable model within each age group when analyzed separately. The primary reason for the lack of fit seems to lie in differential relevance of BIS items for children of different ages, with about 16 items showing modest, but statistically significant negative correlations between item scores and child age. Thus, we do not consider the apparent lack of measurement invariance to threaten the validity of the BIS measure; however, it does suggest that further exploration of age‐specific BI score norms may need to be considered. Further studies of age‐related changes in the BIS are needed to more firmly establish the sensitivity of the BIS to detecting change over time across age groupings.

Even though our sample was similar in proportion to most epidemiological estimates of ASD prevalence in females (e.g., ~20%), we did have a relatively smaller subsample of females. Given previous studies have reported sex differences in ASD in general and RRBs, in particular [Frazier, Georgiades, Bishop, & Hardan, 2014; Sutherland, Hodge, Bruck, Costley, & Klieve, 2017; Szatmari et al., 2012], our findings with respect to sex should be considered preliminary in nature and thus more research on sex influences on inflexibility is warranted. In addition, our sample did not extend below the age of 3 years, and thus more work is needed on potential early manifestations of inflexibility in ASD given that previous studies have reported onset of RRBs in children with ASD as early as 12 months [Wolff et al., 2014]. Finally, we note that 18% of the sample had a score below 12 on the SCQ. This is not surprising given that ratings were obtained on the Current form of the SCQ and that many children were higher functioning according to parent reports.

Of interest, we found that the BIS total score had different associations with different RBS‐R subscales. BI was associated to a greater degree with the insistence on sameness subtype of RRB (*r* = 0.89), and less so with stereotyped (*r* = 0.52) and self‐injurious behavior (*r* = 0.48), although these associations were statistically significant and an order of magnitude higher than the association found between the BIS and social‐communication symptoms as measured by the SCQ. This differential degree of correlation of the BIS with the different RRB subtypes may be worth examining further. This may suggest that the BIS is relatively more sensitive to “higher‐order” types of RRB, such as insistence on sameness, than it is to measuring “lower‐order” types of RRBs such as repetitive body movements. If this is the case, then the BIS likely has clinical relevance because across multiple studies reporting on the factor structure of RRBs in children and adults with ASD, the insistence on sameness factor has consistently emerged [Hus et al., 2007]. This suggests that insistence on sameness may represent a “core” type of RRB that manifests across the spectrum of ASD. If so, then valid measurement of this aspect of RRB and its functional impact may be particularly important in the area of ASD intervention. Alternatively, this may point to a phenomenological aspect of sameness that is conceptually similar to inflexibility but can also manifest in the context of other forms of repetitive behavior such as stereotypy, or rituals/compulsions. In many varieties of RRB, parents and clinicians alike report on the tendency for the person with ASD to become upset when repetitive, habitual patterns of behavior or movement are disrupted or thwarted [Harrop, McBee, & Boyd, 2016; Sethi et al., 2018]. This may indicate that measures of sameness or inflexibility could be measuring the tendency for the person to react when the interest, action, behavior, or movement is changed (i.e., is not kept the same). In this light, scales other than the BIS that measure simply the specific forms of RRB may not be as sensitive to the measurement of a potentially pervasive and underlying core aspect of RRBs in ASD. We note in this context that in the parent and clinician focus groups that we convened for this project, as well as with our expert panel used to review the BIS development process, one common theme that emerged across all groups was that inflexibility or the intolerance of change was viewed by many to be a “sine quo non” or “part and parcel” of ASD. This suggests that inflexibility or insistence on sameness is an appropriate and meaningful treatment target.

Furthermore, the BIS is attempting to measure the functional impact of these behaviors, and thus, is meant to be a more clinically relevant measure. While the RBS‐R and BIS both measure the likely similar constructs of insistence on sameness and inflexibility, the BIS is designed to capture the impact on these behaviors on the child and family and not simply to determine presence/absence of these behaviors. A unique aspect to the BIS is the inclusion of items that capture the manifestation of BI, but also items that consider the impact of BI on the family and family life (Items 10, 14, 18, 25, and 34). These items were developed based on our previous findings from focus groups [Sethi et al., 2018]. Importantly, BIS scores had very high temporal stability, a prerequisite to measuring change [Kraemer, 1992]; although we still need to examine sensitivity to change over longer time periods or with respect to treatment.

Our findings should be considered in light of a few limitations. Using a convenience sample recruited from the internet allowed for a large number of participants, but precludes direct assessments of the children. As such, level of functioning and language ability needed to be estimated by parents and ASD diagnosis could not be confirmed by an expert. Nevertheless, IAN has safeguards in place and these methods are acceptable for initial psychometric analyses. Although this is a reasonable proxy in the context of a large‐scale online survey study, more research is needed to examine the relation of the BIS to cognitive and language ability. Another weakness in our approach to examining convergent validity is that all measures used were parent‐reported measures taken at the same time point and thus shared measurement variance is contributing to our pattern of cross‐item correlations to an unknown degree. To a small extent, the pattern of differential correlations found within our dataset diminishes this concern as parents were apparently able to discriminate different aspects of their child's behavior using the instruments included; however, future studies need to examine the convergent and divergent validity of the BIS using a multimethod approach (e.g., parent reported, clinical interview, direct observation). Establishing validity is an ongoing process and more research on the BIS is needed with samples of children with and without a variety of neurodevelopmental disorders.

In conclusion, preliminary evidence suggests that the BIS is a reliable and valid measure of BI. It also appears to be a promising outcome measure of BI, a relevant symptom domain in ASD, and other neurodevelopmental disorders.

## Conflict of Interest

The authors have no conflict of interests to declare.
